# Effect of Temperature Field and Stress Field of Different Crack Behavior on Twins and Dislocations under Mg Alloy Rolling

**DOI:** 10.3390/ma14195668

**Published:** 2021-09-29

**Authors:** Jing Tian, Jiafei Deng, Quanxin Shi, Yuanying Chang, Wei Liang, Wanggang Zhang

**Affiliations:** 1 College of Materials Science and Engineering, Taiyuan University of Technology, Taiyuan 030024, China; tianjing0033@link.tyut.edu.cn (J.T.); Dengjiafei0116@link.tyut.edu.cn (J.D.); shiquanxin@tyut.edu.cn (Q.S.); changyuanying1063@link.tyut.edu.cn (Y.C.); 2 Shanxi Key Laboratory of Advanced Magnesium-Based Materials, Taiyuan University of Technology, Taiyuan 030024, China; 3 Instrumental Analysis Center, Taiyuan University of Technology, Taiyuan 030024, China

**Keywords:** temperature field, stress field, rolling, dislocation, twinning

## Abstract

Aiming at the problem of the poor plasticity of magnesium alloy leading to serious edge cracks in the rolling process, this paper conducts a systematic study on the crack suppression mechanism of rolling under different thickness reductions. Using restricted rolling and conventional rolling, comparing the microstructure evolution of the plate after rolling, and combining the information of the simulated temperature field and stress field of the plates, the behavior of twins and dislocations under different thickness reductions is explained, and the influence of serious damage caused by single-pass hot rolling of magnesium alloy is explored. The compressive stress fields along with the transverse and normal directions under restricted rolling cause the compression twins to mature into secondary twins under rolling with small thickness reduction and induce a large number of tensile twins when the thickness reduction amount is increased. The multiple slips activated by the higher temperature field at the edge of the small thickness reduction amount cause dislocations to be distributed inside and outside the twins, while the edge with large thickness reduction can activate more slip due to the high-temperature field resulting from friction, resulting in the twin be destroyed.

## 1. Introduction

As the lightest structural metal of the 21st century, magnesium alloy is widely used in the automotive and aerospace fields. Among them, magnesium alloy rolled sheet is a common material for complex components in the industry, and the improvement of its production efficiency has attracted the attention of researchers [1,2,3,4,5]. The cracks of Mg alloys, after being hot rolled under a single-pass rolling with large thickness reductions, are seriously damaged and difficult to solve due to too much deformation [6,7,8].

Researchers have explored this problem. Briffod et al. [9] found that basal slip and deformation twinning due to its low critical resolved shear stress (CRSS) and hardening rate are the main deformation mechanisms under uniaxial complete reverse fatigue conditions. Through the simulation of tension rolling and the analysis of the stress state of the rolling slip zone, Ning et al. [4] reduced the rolling force by applying tension, thereby reducing friction and suppressing the cracking tendency of the plate. Jia et al. [10] found that the edge crack fracture mode of the longitudinal composed of RD (rolling direction) and ND (normal direction) section of the rolled sheet is dominated by 45° cross-shear cracks. As the rolling temperature decreases and the thickness reduction increases, the crack depth, along with the TD (transverse direction), increases. Kun et al. [11] found that the local strain unevenness caused by rapid extrusion and the difference in plastic deformation capacity have an important impact on the cracking of the composite extruded sheet, and the non-basal texture can inhibit crack propagation. 

Even though there are many types of research on cracks [12,13,14], effective solutions to cracks have not yet been explored, and there are few studies on the mechanism of effectively suppressing the edge cracks of magnesium alloys during rolling.

Restricted forming positively affects the formability of a Mg alloy, like the entire die forging, and severe plastic deformation (SPD) methods [15]. The material undergoes severe plastic deformation to greatly refine the grain structure and obtain good plasticity. Restricted forming can also be applied to magnesium alloy rolling to solve the problem of serious damage caused by single-pass hot rolling of magnesium alloy with large thickness reduction. This paper will use restricted rolling through grooves of the same width as the plate on the roller to restrict the widening deformation of the magnesium alloy plate during rolling to bear severe plastic deformation so that it can obtain better plasticity and effectively suppress and inhibit the generation of edge cracks in the hot rolling process of the magnesium alloy in a single-pass rolling, which is of great significance for continuous and efficient production of magnesium alloy thin sheets.

This paper will use restricted rolling to simulate the stress field and temperature field, study the microstructure evolution of the plate under the two rolling methods and compare the force field and temperature field of the plate in conventional rolling. In this research, the evolution of the mechanism to improve serious damage during rolling is explored and an effective mechanism for solving the problem of serious damage caused by single-pass hot rolling of magnesium alloy is proposed.

## 2. Material and Methods

**Figure 1:**
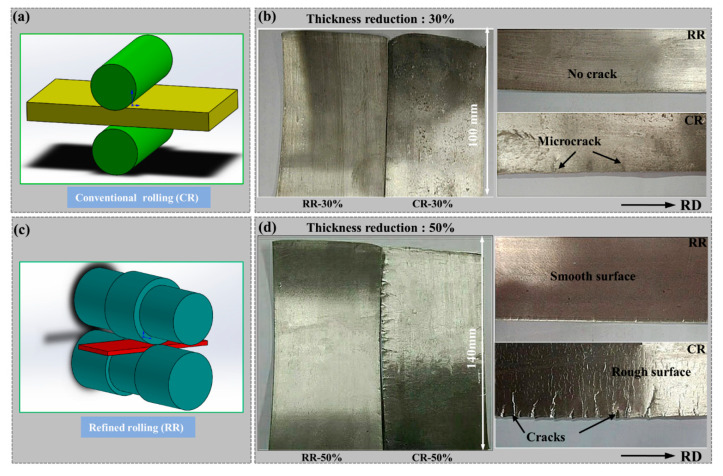
(**a**,**c**) Schematic diagram of the rollers and (**b**,**d**) crack behavior.

The 3 mm thick AZ31 magnesium alloy plate was subjected to conventional rolling and restricted rolling at 400 °C, as shown in Figure 1. The original plate had a length of 70 mm in RD and a length of 60 mm in the TD. Roll the plate to RD of 100 mm and 140 mm in one pass rolling to obtain CR30% (conventional rolling, the thickness reduction is 30%), CR50% (conventional rolling, the thickness reduction is 50%), RR30% (restricted rolling, the thickness reduction is 30%), and RR50% (restricted rolling, the thickness reduction is 50%) to study the suppression of micro-cracks at small thickness reduction and severe cracks at large thickness reduction. The 4 mm (RD) × 8 mm (TD) samples were cut from the plates (CR30%, CR50%, RR30%, RR50%), using an optical microscope (OM; Leica–2500M, Leica, Germany), a field emission scanning electron microscope (SEM; TESCAN Mira 3, TESCAN, The Czech Republic) equipped with an electron backscatter diffraction (EBSD) system and Aztec software (Oxford Instruments, Oxford, England) and the transmission electron microscope (TEM; JEM 2100F, JEOL, Tokyo, Japan) to observe the microstructure evolution. The samples were grinding on 400, 800, 1200, 1500, 2000 and 3000 grit SiC paper and then electro-polishing at a voltage of 25 V and an electric current of 0.30 A for 90 s at a temperature of −20 °C. The step size, working distance, and voltage of EBSD are 0.4 μm, 20 mm, and 20 V, respectively. The 20 mm (RD) × 15 mm (TD) samples were cut from the plates and their macrotexture was observed on the Rigaku Smart Lab SE diffractometer (Rigaku Smart Lab, Rigaku Corporation, Tokyo, Japan). X-ray diffraction (XRD) was performed using CuKα radiation at a scanning speed of 5°/min and a step size of 5°. The rolling processes of CR30%, CR50%, RR30%, RR50% plates were simulated by using Abaqus software, and the temperature field and stress field were explored.

## 3. Results and Discussion

### 3.1. Crack Behavior

Figure 1b,d present the cracks seen in the plate after rolling with large reduction and small reduction. When the reduction is small, a slight crack appears at the edge of the conventional rolling, and the depth is about 5 mm. Severe cracks appear when the reduction is large, and the depth is about 10 mm. However, the rolled plate is restricted to always maintain a smooth surface and a crack-free state. Slight cracks under small reductions and severe cracks under large reductions have different suppression mechanisms. Studying the evolution of the mechanism of suppressing cracks is of great significance for improving the rolling forming rate of magnesium alloys.

### 3.2. Microstructure Evolution

**Figure 2:**
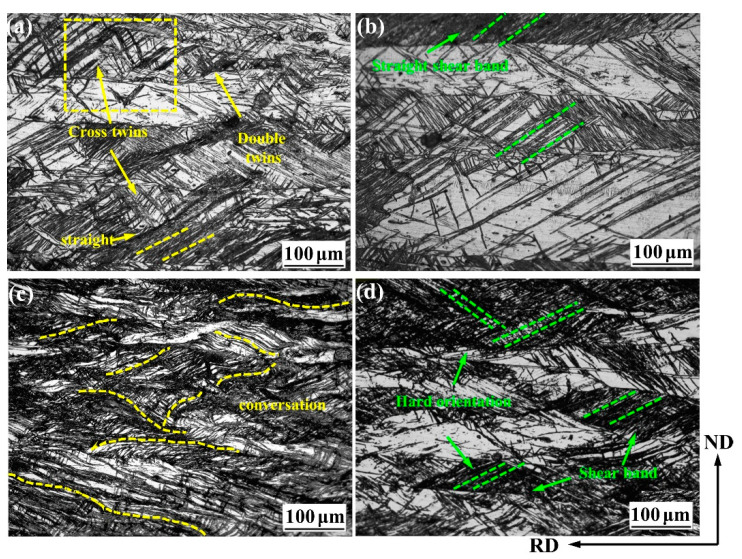
Metallographic microstructure of (**a**) CR30%, (**b**) RR30%, (**c**) CR50%, and (**d**) RR50%.

Figure 2 is the metallographic microstructure of CR30%, CR50%, RR30%, and RR50% plates. When the thickness reduction is 30%, the shear band of conventional rolling and restricted rolling are dominated by the straight shear bands, which are typical rolling morphologies. The difference is that restricted rolling has more cross twins and secondary twins. When the thickness reduction is increased to 50%, the change in conventional rolling is that the shear band becomes denser, and the unevenness of the microstructure is greatly improved. The uneven microstructure will lead to stress concentration, which is very likely to cause cracks. In addition to being denser, the shear band of restricted rolling has its shape changed from straight to corrugated. This microstructure evolution improves the stress concentration caused by the dense shear band in conventional rolling, and the microstructure is relatively evenly distributed, resulting in the probability of cracks being small.

**Figure 3:**
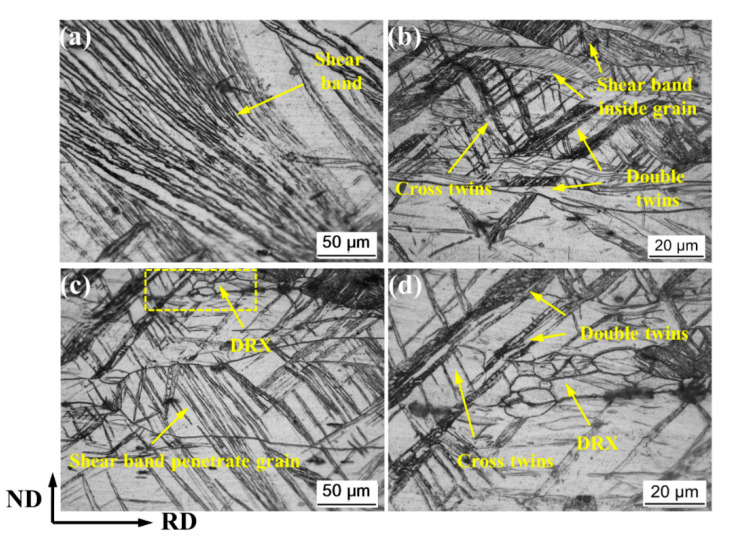
Detailed metallographic microstructure of RR30%: (**a**) shear band, (**b**) twins and shear band, (**c**) shear band penetrate grain, and (**d**) recrystallization.

Figure 3 is a metallographic diagram of the local organization of RR30% and RR50%. When the thickness reduction is 30%, there are clearly visible shear bands (Figure 3b) and a slightly corrugated tendency, and it is distributed in a wide range in the microstructure of the rolled plate. The relationship between shear bands and grains can be roughly divided into a shear band wrapped by crystal grains and a shear band penetrating the grain boundary, as shown in Figure 3a,c. In addition, a large number of secondary twins and cross twins are generated, which can easily become nucleation sites for recrystallization, as shown in Figure 3c. Figure 3d is an enlarged image of the yellow dashed frame in Figure 3c, showing the recrystallization formed at the cross twins.

**Figure 4:**
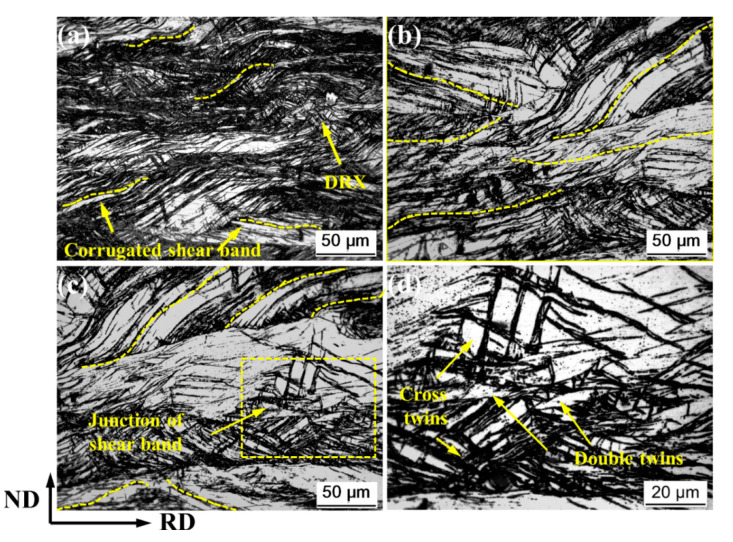
Detailed metallographic microstructure of RR50%: (**a**,**b**) shear band, (**c**) junction of shear band, and (**d**) twins.

When the deformation is increased to 50% of the thickness reduction, the shear band becomes denser, and the width-limited rolling will cause the shear band to undergo a significant morphological change, which becomes corrugated shear bands, as shown in Figure 4. The appearance of corrugated shear bands homogenized the microstructure to a large extent, and the stress concentration situation was effectively improved. It is very easy to produce dynamic recrystallization due to larger deformation. At the junction of the shear bands, a large number of secondary twins are produced, as shown in Figure 4d.

**Figure 5:**
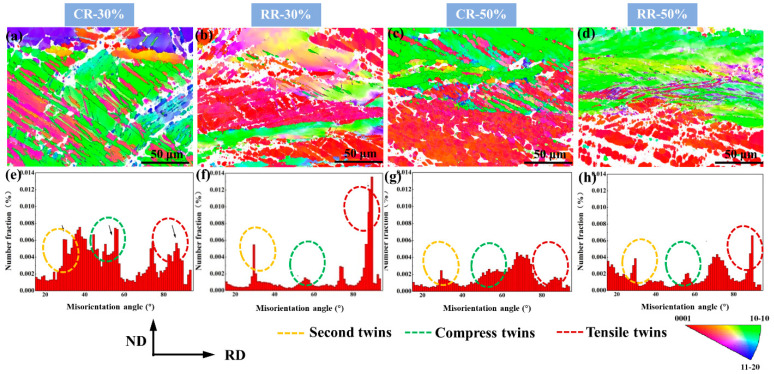
(**a**–**d**) The inverse pole figure and (**e**–**h**) the grain misorientation distribution diagram of CR30%, CR50%, RR30%, and RR50% plates.

Figure 5 shows the inverse pole figure and the grain misorientation distribution diagram of CR30%, CR50%, RR30%, and RR50% plates. From the inverse pole figure, CR30% and CR50% plates both show a straight compression twin morphology, and from the distribution of grain misorientation, they both have obvious peaks near 56°, thus compression twins are most likely to be produced under conventional rolling, and as the reduction increases, the compression twins become thinner [16].

The twins of RR30% and RR50% are relatively abundant. Both the tensile twins and the secondary twins have obvious peaks, and it is easy to produce tensile twins due to the pressure perpendicular to the c-axis of the crystal grains. Comparing the grain misorientation distribution diagram, it can be seen that no matter the thickness reduction amount, the most common twin is the compression twin under conventional rolling. The difference is that under large thickness reductions, more dislocations due to larger deformation will lead to an increase in the content of sub-grain boundaries, and the increase of dislocations in twins will cause the twins to be destroyed, and the twin content is correspondingly reduced. Similarly, RR50% contains more sub-grain boundaries than RR30% due to larger deformation.

**Figure 6:**
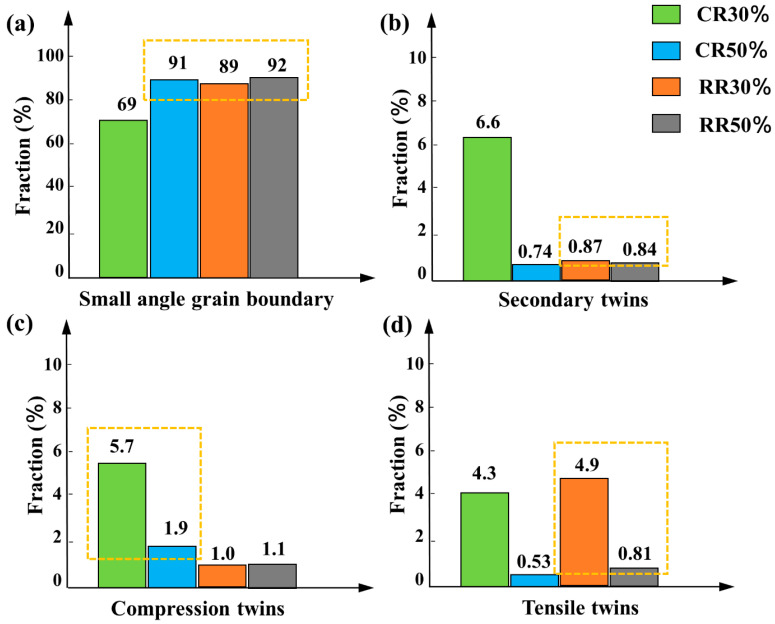
The content of (**a**) small-angle grain boundaries and (**b**–**d**) grain boundaries with three kinds of twin orientations of the CR30%, CR50%, RR30%, and RR50% plates.

Figure 6 shows the content of small-angle grain boundaries and grain boundaries with three kinds of twin orientations of the plates, which can indirectly reflect the content of three twins and the content of dislocations. It can be seen from Figure 6 that it is basically consistent with the conclusions obtained in Figure 5. In addition, although there are more secondary twins in conventional rolling, there are still more compression twins, and there are more secondary twins in restricted rolling and a very small amount of compression twins, thus compression twins in restricted rolling tend to mature into secondary twins. With the increase of the thickness reduction, the content of different kinds of twins is less. This is because the existence of dislocations disrupts the growth of twins, and the deformation is large and difficult to resolve.

**Figure 7:**
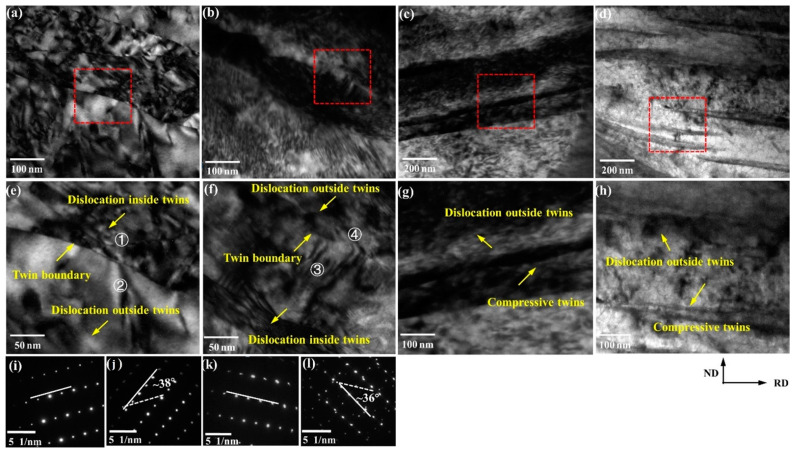
The content of small-angle grain boundaries and grain boundaries with three kinds of twin orientations of the (**a**,**e**) CR30%, (**b**,**f**) CR50%, (**c**,**g**) RR30%, and (**d**,**h**) RR50% plates. (**i**–**l**) The diffraction patterns taken from the region 1, 2, 3 and 4.

In order to further observe the interaction between twins and dislocations, we used TEM to characterize the microstructure evolution. Figure 7 shows the TEM results of CR30%, CR50%, RR30%, and RR50% plates. Figure 7e–h are enlarged views of the red dashed box in Figure 7a–d. Figure 7a,b, respectively, show the plates after the restricted rolling with large thickness reduction and small thickness reduction. The twins have a certain width and there are many dislocations inside and outside the twins. The difference is that there is no obvious twin boundary in twins under large thickness reductions. Figure 7i–k,l are taken from zone 1 and zone 2 in Figure 7e and zone 3 and zone 4 in Figure 7f, respectively. It can be seen that they are all secondary twins. After conventional rolling, they all show slender compression twins, which is same as the twins in Figure 5a. As the thickness reduction increases, the compression twins become shorter in Figure 5a,c. This is due to the destruction of compression twins under the action of dislocations outside the twins.

**Figure 8:**
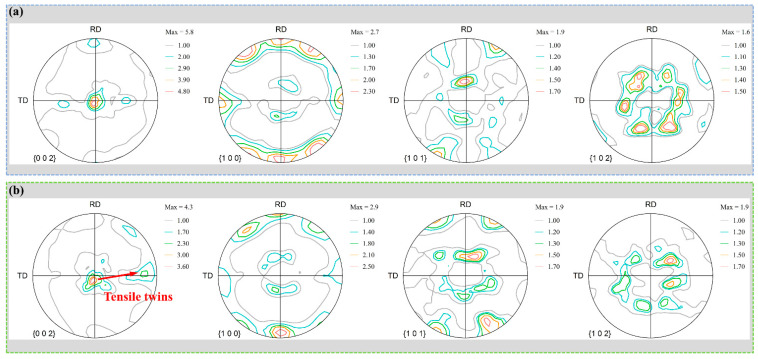
The macrotexture of (**a**) CR30% and (**b**) RR30%.

Weakening basal texture is an effective measure to improve the formability of magnesium alloys [17]. Due to the conventional rolling under large thickness reduction causes serious edge cracks on the edges of the plate in Figure 1, it will cause serious errors in the measurement of the macrotexture. Therefore, here only the conventional rolling plate under a small thickness reduction is compared to the plate after restricted rolling with the same position, and the crack of the plate under conventional rolling is removed as shown in Figure 8. Due to the introduction of tensile twins in Figure 5b,d, restricted rolling has weakened the basal texture. Compared with conventional rolling, it is more conducive to coordinated deformation, thus cracks are less likely to occur in Figure 1. 

**Figure 9:**
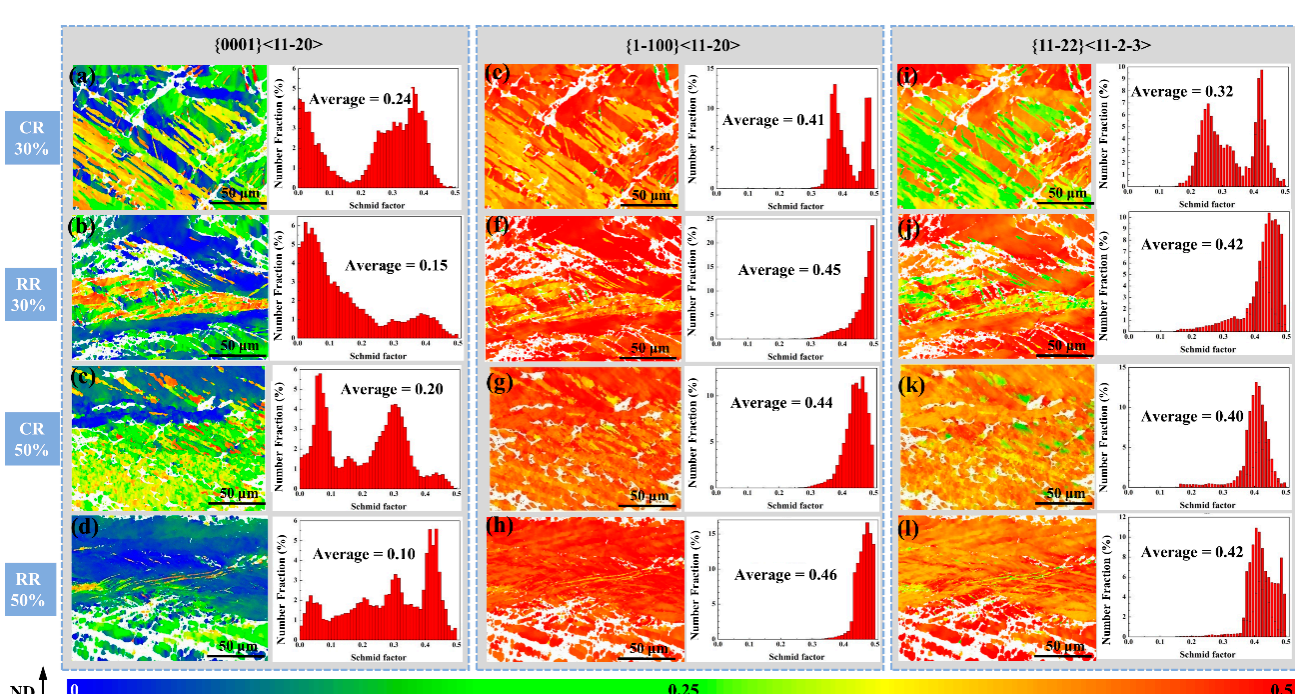
The Schmidt factor value of (**a**,**e**,**i**) CR30%, (**b**,**f**,**j**) CR50%, (**c**,**g**,**k**) RR30%, and (**d**,**h**,**l**) RR50% plates.

At the same time, the Schmidt factor distribution chart of CR30%, CR50%, RR30%, and RR50% shown in Figure 9 also shows that the microstructure after conventional rolling is in ‘hard orientation’, which is not conducive to coordinated deformation. Since the cracking is a tensile force in the RD, the loading direction is set to RD.

The Schmidt factor value of the basal slip under conventional rolling is higher than that of the restricted rolling because the straight and slender compression twins have a larger Schmidt factor of basal slip. The slip of the base surface cannot coordinate the deformation of the c-axis, thus it cannot effectively coordinate the deformation and suppress the occurrence of cracks. The Schmidt factor values of prismatic slip and pyramidal slip of RR30% and RR50% are larger. Among them, the slip can effectively coordinate the deformation of the c-axis, so that the plate is not easy to crack after the width-limited rolling.

### 3.3. Stress Field and Temperature Field

In order to explore the influence of plate stress on plate structure, the stress field and temperature field of the plate are studied separately. We used Abaqus finite element simulation software to simulate the stress field and temperature field in the rolling process of CR30%, CR50%, RR30%, and RR50% plate; to compare the changes of stress distribution and temperature distribution; and to explore the reason that cracks of conventional rolling occur and the reason why restricted rolling suppresses cracks.

Abaqus is used to establish the part of the rollers and the magnesium alloy sheet. The rollers are set as a rigid body and do not participate in the analysis. The magnesium alloy sheet gives the material properties. The parameters are shown in Table 1.

Due to the temperature field analysis, in addition to material elasticity and plasticity, thermodynamic parameters such as inelastic heat fraction, specific heat, and conductivity need to be set. The inelastic heat fraction is set to 0.9. Among them, specific heat and conductivity are affected by temperature, as shown in Equations (1) and (2) [18,19,20].

(1) $$C_{\mathrm{PMg}}=991.3863+0.0098\times T+{0.0004\times T}^{2}+({1.78\times 10}^{-7})\times T^{3}$$

(2) $$K_{\mathrm{Mg}}=8.4294+0.3417\times T-{0.0004\times T}^{2}+({2.2592\times 10}^{-7}){\times T}^{3}$$

The analysis step selects ‘Dynamic, Temp-disp, Explicit’, and the time is set to 6 s to ensure that the plate passes through the roll successfully. General contact (explicit) is selected for contact, and the friction behavior between the contact surfaces is defined by Coulomb’s law of friction, and the friction coefficient is 0.5. Set the surface film condition: the film coefficient is 0.02 and the sink’s temperature is 293 K. The roll speed is set to 10 rad/s and the initial temperature of the plate is 693 K.

To ensure the accuracy of the calculation, the plate grid is mashed into 112,000, and the couple temperature-displacement is selected. Using the Lagrangian analysis method, Von-Mises stress is used to define the equivalent stress, as in Equation (3) [21,22], $\sigma_{1}$, $\sigma_{2}$, and $\sigma_{3}$ are used to represent the principal stress. $\sigma_{1}$, $\sigma_{2}$, and $\sigma_{3}$ correspond to the max principal, mid principal, and min principal, respectively. These three quantities are invariants in any coordinate system.

(3) $$\bar{\sigma }=\frac{1}{\sqrt{2}}\sqrt{{\left(\sigma_{1}{-\sigma }_{2}\right)}^{2}+{\left(\sigma_{3}{-\sigma }_{2}\right)}^{2}+{\left(\sigma_{1}{-\sigma }_{3}\right)}^{2}}$$

**Figure 10:**
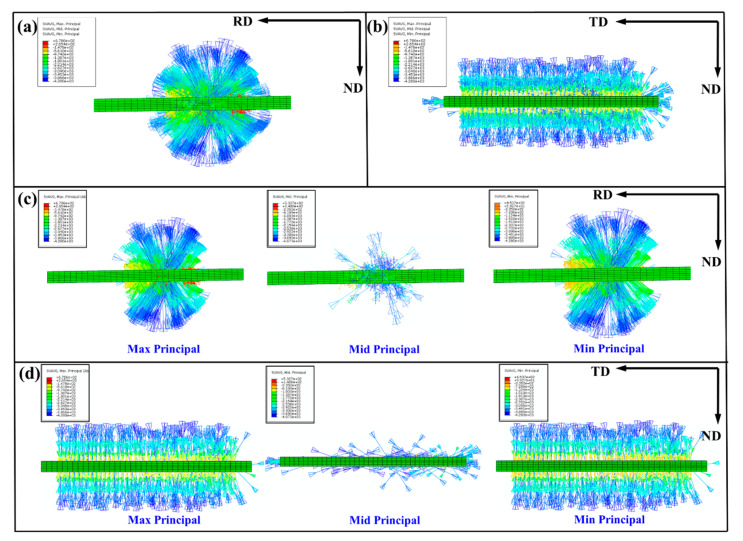
Analysis of max principal, mid principal, and min principal of RR50% on the (**a**,**c**) RD-ND plane and (**b**,**d**) TD-ND plane.

Figure 10 is the analysis of max principal, mid principal, and min principal of RR50%. Because the rolling force is perpendicular to the arc of the roll, the force of the plate is in all directions at the time of rolling, and it appears spherical on the RD and ND surfaces, as shown in Figure 10a. Figure 10c shows that the stress distribution is mainly caused by max principal and min principal. From Figure 10b, it can be seen that the plate is subjected to lateral pressure, which is mainly caused by the mid principal, as shown in Figure 10d. Additionally, the lateral pressure leads the formation of tensile twins in Figure 5b,d.

**Figure 11:**
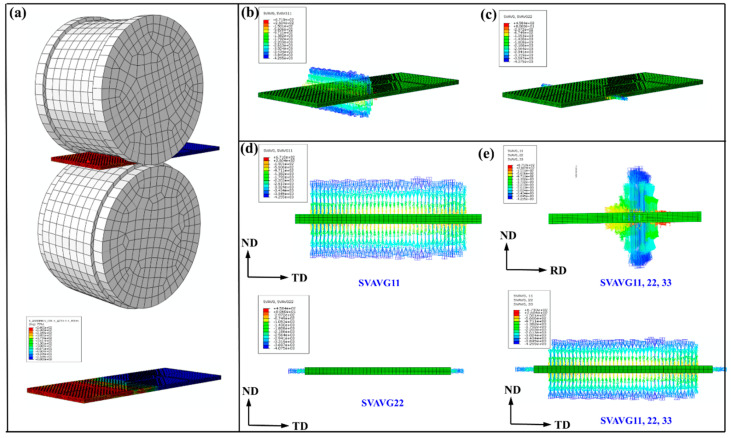
(**a**) Models and (**b**–**e**) the average stress per unit volume of RR50%.

Figure 11 is the average stress per unit volume. Figure 11b,c, respectively, show the pressure of the plate in the normal direction (ND) of the roll during the rolling process and the pressure of the plate in the transverse direction (TD) caused by the shape of the rollers. Figure 11e can more clearly show the overall stress distribution of the plate under restricted rolling.

**Figure 12:**
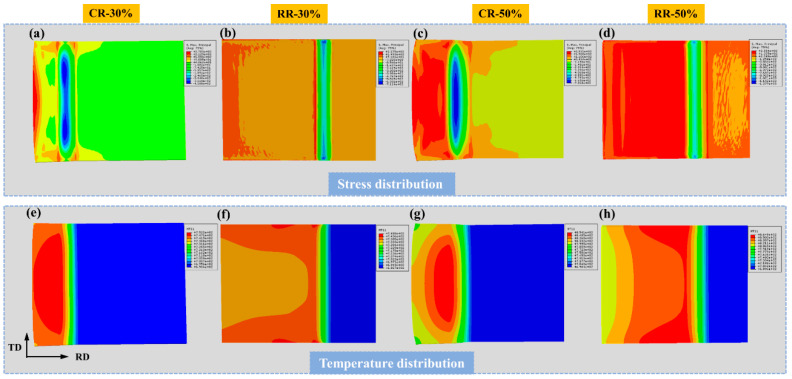
The stress distribution and temperature distribution of (**a**,**e**) CR30%, (**b**,**f**) CR50%, (**c**,**g**) RR30%, and (**d**,**h**) RR50% plates.

Figure 12 is the force distribution and temperature distribution of CR30%, CR50%, RR30%, and RR50%. During conventional rolling, both sides of the plate are subjected to obvious tensile force, which leads to the generation of cracks. Restricted rolling evens out the stress distribution. The different stress distribution influences grain orientation, leading different kinds of twins in EBSD results.

In addition, the heat dissipation of conventional rolling is more obvious, and the decrease in temperature leads to lower plasticity of the plate and easier cracking. The temperature distribution of the rolled plate with restricted rolling illustrates that the edge of the plate has a higher temperature due to the friction between the rollers and the plate, which helps to ensure the plasticity of the edge of the plate to a certain extent, and is beneficial to suppress the generation of cracks in Figure 1.

**Figure 13:**
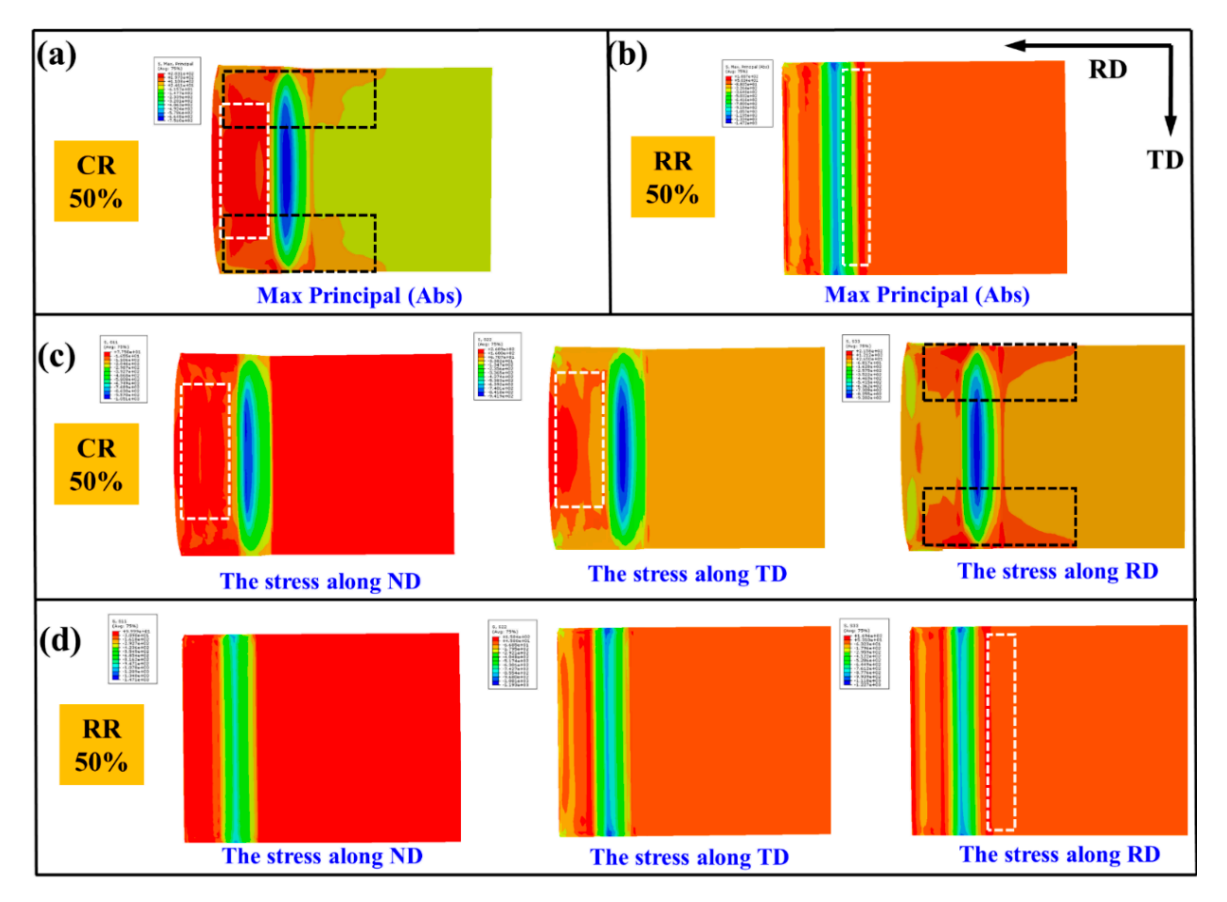
Abaqus analysis of the stress distribution of the (**a**,**c**) CR50% and (**b**,**d**) RR50% plates.

Figure 13 is the Abaqus stress analysis of CR50% and RR50% rolled plates. Figure 13a,b are the max principal distribution of CR50% and RR50%. The large tensile stress along the RD at the edge of the plate leads the edge and the center of the plate to be asynchronously extended [23], resulting in edge cracks along the TD [24]. For the CR50% plate, the main stress at the center of the plate shown in the white dotted frame (Figure 13a) is mainly provided by the stress along with ND and TD in the white dotted frame (Figure 13c), while the main stress at the edge of the plate shown in the black dotted frame is mainly provided by the stress along RD, causing the crack to expand along with TD. The max principal of the RR50% plate in a white dotted frame (Figure 13b) is caused by the stress along RD in a white dotted frame (Figure 13d). 

### 3.4. The Effect of Temperature Field and Stress Field on Microstructure Evolution

**Figure 14:**
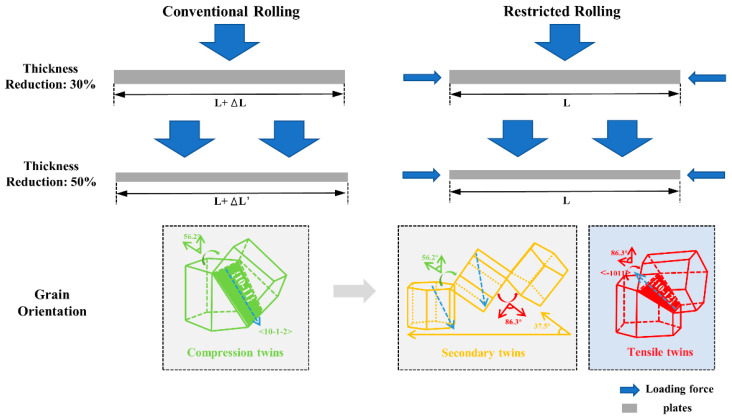
The relationship between grain orientation and stress.

Figure 14 shows the change of grain orientation when the plate is stressed under this force. During conventional rolling, the plate is only subjected to pressure along with ND in Figure 13a. At this time, under high pressure, the grains are easily rotated around the c-axis by 56°, that is, compression twins are produced in Figure 5b. When the thickness reduction amount becomes larger, that is, when the pressure becomes larger in Figure 13c, more compression twins are produced in Figure 5c. The stress distribution of the sheet during conventional rolling affects the morphology of the compression twins in the EBSD results.

Under restricted rolling, in addition to the pressure along the ND, the plate also has the pressure along the TD. The pressure along ND in Figure 11d leads to the generation of compression twins, and the superposition of the pressure along TD in Figure 11d leads to the re-growth of twins, that is, when the compression twins continue to rotate 86° around the c-axis, compression-tension twins are produced, and the compression twins mature into secondary twins in Figure 5b. Since the pressure along TD is perpendicular to the c-axis of the grains, tensile twins are extremely easy to produce. Compared with conventional rolling, the more complicated stress distribution of restricted rolling leads to different changes in the grain orientation, and the types of twins are more abundant.

**Figure 15:**
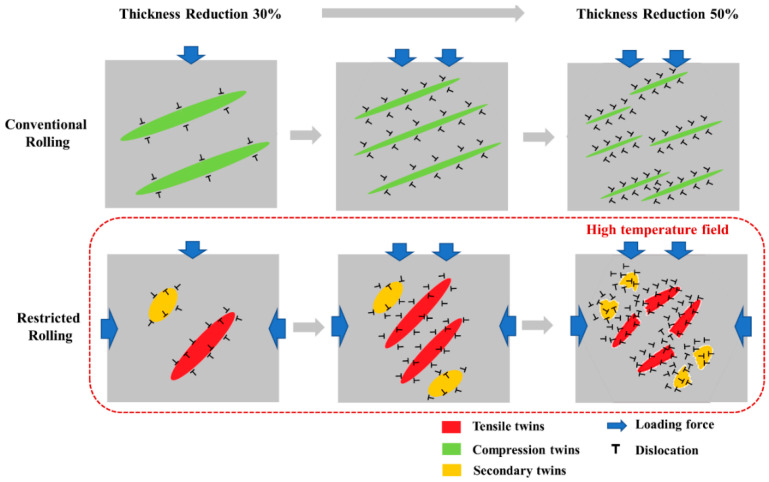
The coordination of twins and dislocations.

Figure 15 shows the coordinated effect of twins and slips. Conventional rolling has fewer dislocations at a small thickness reduction and surrounds the compression twins. When the deformation increases, the compression twins become finer, and the dislocations increase sharply. The dislocations surrounding the twins destroy the compression twins, thus the twins become shorter and thinner.

However, due to the more complex stress field during restricted rolling, there are more dislocations because high temperature leads to more slip activated, which are distributed inside and outside the secondary twins and tensile twins. When the deformation increases, the twins are easily damaged by the dislocation distributed inside and outside twins. The twins are destroyed to a certain extent, and the structure is uniform, which is beneficial to coordinating strain.

The increase of dislocations in conventional rolling is only due to the large deformation caused by the large thickness reduction. The increase of dislocations under restricted rolling is mainly caused by two reasons: (1) The increase of the thickness reduction, and the complex stress of the plate during restriction rolling causes it to bear greater plastic deformation. (2) Due to the friction between the plate and the roller, the edge of the sheet has a higher temperature, which will cause more slippage systems to be activated. The greater the thickness reduction, the higher the temperature of the edge of the sheet due to friction.

In summary, the stress field and temperature field of the sheet during the rolling process will affect the change of the grain orientation, leading to the generation of different twins. In a higher temperature field, it is easier to activate slip, leading to the accumulation of dislocations, thereby affecting the interaction between twins and dislocations.

## 4. Conclusions

1. Compression twins that are easily produced during magnesium alloy rolling are mostly elongated. The increase in thickness reduction increases dislocations and destroys the compression twins.
2. The stress field of restricted rolling is composed of the rolling force and the transverse pressure of the plate. The rolling force causes the generation of compression twins, and the lateral pressure causes the compression twins to mature and grow into secondary twins. At the same time, the grains are compressed perpendicular to the c-axis, resulting in the generation of tensile twins. A large number of tensile twins are induced when the thickness reduction amount is increased.
3. With the increase of the thickness reduction, the higher temperature field that restricts the edge of the rolled plate leads to the tensile twins and the secondary twins are easily broken, which is beneficial to the uniformity of the structure and the coordinated stress distribution, thereby reducing the tendency of cracking.

## Figures and Tables

**Table 1:** Material properties of AZ31B used for Abaqus simulation.

Density	Elongation	Young’s Modulus	Poisson Ration	Tensile Strength	Yield Strength
1780 kg/m^3^	16.4%	52,479.23 MPa	0.34	260 MPa	87 MPa

## Data Availability

The data that support the findings of this study are available from the corresponding author upon reasonable request.
